# Rapid Separation of Indole Glucosinolates in Roots of Chinese Cabbage (*Brassica rapa* Subsp. Pekinensis) by High-Performance Liquid Chromatography with Diode Array Detection

**DOI:** 10.1155/2017/5125329

**Published:** 2017-06-13

**Authors:** Alfredo Aires, Rosa Carvalho

**Affiliations:** ^1^Centre for the Research and Technology for Agro-Environment and Biological Sciences (CITAB), Universidade de Trás-os-Montes e Alto Douro (UTAD), Quinta de Prados, 5000-801 Vila Real, Portugal; ^2^Agronomy Department, Universidade de Trás-os-Montes e Alto Douro (UTAD), Quinta de Prados, 5000-801 Vila Real, Portugal

## Abstract

Glucosinolates are a class of sulphur-containing plant compounds with diverse biological properties. They have been found exclusively in the Brassicaceae family plants and studied exhaustively in biosynthetic and application perspectives. The aim of this current study is to present a simple and updated method to quantify indole glucosinolate content in hairy root cultures of Chinese cabbage by HPLC-DAD-UV/Vis. Method validation controls were performed and recovery experiments were assayed. The data was statically treated and compared with published works. The current method allowed a feasible identification of indole glucosinolates and it was possible to identify accurately three indole glucosinolate compounds (glucobrassicin, 4-methoxyglucobrassicin, and 1-methoxyglucobrassicin) in roots of Chinese cabbage. The method demonstrated a good linearity (*R*^2^ > 0.99), a good precision, and selectivity sensitivity. In conclusion, this protocol provides an accessible method to extract and quantify glucosinolates in plant samples. Thus, based on our results, the method is valid and can be extended to other plant or food matrices.

## 1. Introduction

**Figure 1:**
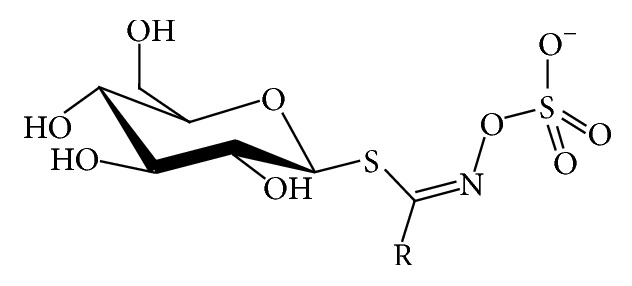
General structure of glucosinolates. Adapted from Griffiths et al. (1998) [4].

Glucosinolates (GLS) are well-known secondary metabolites class type, largely present in Brassicaceae family plants, like Chinese cabbage, Brussel-sprouts, kale, cauliflower, broccoli, turnips, colza, and all types of cruciferous plants. Until now, about 200 different GSLs have been identified and characterized in wide groups of plants [1, 2], including at least 40 different kinds of* Brassica* vegetables [3]. There are different types of GLS sharing a common chemical structure (Figure 1) composed of *β*-thioglycoside N-hydroxysulfates, with a side chain R and a sulphur linked *β*-D-glucopyranose moiety [4], and depending on the primary amino acids they can be divided into aliphatic (derived from Ala, Leu, Lle, Val, and Met), aryl (derived from Phe or Tyr), and indole (derived from Trp.). They occurs simultaneously with an endogenous enzyme myrosinase (EC3.2.1.147), which can decompose them into several hydrolysis products when in contact with each other (by freezing injury, sunburn, chewing, cell walls lysing, pest attacks and diseases, and several other injuries during harvesting or during plant grow season) [5, 6]. Depending on the reaction conditions like GLS side chain structure, cell pH, presence of cofactors (Fe^2+^ and proteins), and myrosinase conditions, the resulting glucosinolate hydrolysis products (GHP) can be isothiocyanates (ITCs), nitriles, thiocyanates, epithionitriles, and oxazolidinethiones [4, 7–10]. Among these, ITCs are considered the most powerful GHP, since important associations between GHP and activities with beneficial effects on human health were found, such as antimicrobial, antitumoral, antimutagenic, anti-inflammatory, and anticancer activities [8, 11–20]. Different analytical methods, which have been developed to quantify GLS through HPLC, NMR, ELISA, mass spectroscopy, biosensing, and near-infrared spectroscopy, have been developed. Nonetheless, they present different approaches and some of them are very expensive, time consuming, and limited in usage for a routine analysis. Moreover, some of them are less clear about their analytical calculations such as response factors. Therefore, this paper aims to present a simple, feasible, and validated method to extract and quantify indole GLS in plant samples, in particular in Chinese root cabbages. However, the results can be extended to other types of plant material.

## 2. Experimental

### 2.1. Sampling

Three types of samples were used in this study: (i) hairy roots of* Brassica rapa* subsp. pekinensis, (ii) the GL standard of sinigrin (Sigma-Aldrich, Taufkirchen, Germany), and (iii) Gl standard of glucotropaeolin (benzyl GL) (ceded by Professor of Eduardo Rosa from University of Trás-os-Montes e Alto Douro, Vila Real, Portugal and Dr. Renato Iori from Istituto Sperimentale Colture Industriali Bologna, Italy). Hairy roots of* Brassica rapa* subsp. pekinensis were ceded by Madeleine Neumann and Jutta Ludwig-Müller from Technische Universität of Dresden, Dresden, Germany. These hairy cultures were grown for 18 days in MS medium, and after this period, the roots were harvested, dried, and milled in ultrafine powder in a commercial blender. These 3 types of samples were used to extract and quantify GLS.

### 2.2. Extraction of GLS

The extraction method was based on the previous methods of International Standard Method ISO 9167-1 [22] and European Commission [21], but with several modifications. For extraction, 200 mg dry weight (dw) of each sample was added to 5 mL 70% methanol : water and heated at 70°C for 10 minutes and then centrifuged at 4000 rpm for 20 minutes. The supernatants were collected and the final volume was adjusted with methanol 70% to a final volume of 10 mL. The extracts were submitted to an enzymatic desulfation of GLS.

In desulfation process, each methanolic extract with crude GLS was loaded onto a DEAE Sephadex A-25 anion exchange column, which was prepared by adding 0.5 mL DEAE Sephadex A-25 (preequilibrated with 0.5 M acidic pyridine [20 mL pyridine, 15 mL of acetic acid, 465 mL water] and 0.02 M of acidic pyridine [0.8 mL pyridine, 0.6 mL acetic acid, 498.6 mL of water]) to empty fritted SPE tubes (SUPLECO, ref^a^ 54220-U, Belafonte, USA). Firstly, after adding 0.5 mL of preequilibrated resin, each column was washed with 0.5 mL of ultrapure water to remove cation and neutral ions; then 2 mL of each sample was loaded onto columns and washed twice with 1 mL of ultrapure water, followed by addition of 0.5 mL pyridine 0.02 M. Then 75 *μ*L of aryl sulfatase (E.C.3.1.6.1) type H1 from* Helix pomatia* (Sigma-Aldrich, Taufkirchen, Germany) was loaded into each column, followed by a desulfation reaction overnight (16–18 hours) at room temperature. The desulfated GLS were then eluted with 0.5 mL (×3 times) of ultrapure water. The eluates were stored at −80°C prior to HPLC analysis.

The enzyme sulfatase was prepared adding 140 mg of pure sulfatase (Sigma-Aldrich, Taufkirchen, Germany) in 12 mL of 50% aqueous ethanol followed by centrifugation at 4000 rpm for 5 minutes. The supernatant was collected and precipitate rejected. The supernatant was diluted with 1.5 equal volume of water. This mixture was centrifuged again at 4000 rpm for 5 minutes. Now the supernatant was rejected and the precipitate diluted with 4 mL of ultrapure water. This final volume was then loaded in a column of 1 mL Sephadex A25. The 4 mL was loaded into a column of 1 mL of A25 followed by loading into a column of 1 mL Sephadex C25. The final eluate was collected and used in the samples for desulfation of GLS.

### 2.3. Determination of GLS by HPLC

The extracts with desulphoglucosinolates were injected in a HPLC-DAD-UV/Vis equipped by a C18 (250 × 4.60 mm, 5 *μ*m) column with a mobile phase of ultrapure water (solvent A) and 200 mL L^−1^ acetonitrile : water (v/v) (solvent B), with a flow rate of 1.5 mL min^−1^ and an injection volume of 10 *μ*L, with a binary gradient of 1% of solvent B at 1 minute, 99% of solvent B at 21 minutes, 99% of solvent B at 24 minutes, 1% of solvent B at 29 minutes, and 1% of solvent B at 39 minutes. The full length run was 39 minutes. The GSs peak identification and quantitative estimations were made using pure standard GSs as internal standard (IS) (benzyl GL at 1 mg mL^−1^). The chromatograms were recorded at 229 nm and used to identify GSs by retention time (RT) and UV spectra in comparison with commercial standards. All reagents used in analytical determinations were HPLC grade.

### 2.4. GLS Quantification

The quantification of GLS was based on the internal standard method, according the fundamentals of HPLC widely accepted in experimentation supported by the guidelines of International Conference on Harmonization (ICH) [24]. The following formula was used to quantify each GL content as *μ*moles·100 g^−1^ dry weight (dw): *C*_GLS  sample_ = (*C*_is_ × HPLC  Area_GLS  sample_ × Rf × Df)/(HPLC area is × dw) × 100, where *C*_GLS  sample_ is the concentration of each GL in sample; *C*_is_ is the concentration in *μ*moles·mL^−1^ of internal standards added to sample and injected in the HPLC, Rf is the response factor of each GLS, and Df is the dilution factor. The Rf factors, for each GLS, detected at 229 nm, are already published in different papers and are listed in Table 1. In the situation of undefined GLS, the Rf should be considered 1, following the convention for similar situations.

### 2.5. Method Validation and Analytical Quality Assurance

Calibration curves for glucotropaeolin and sinigrin, with 7 points each, were established in the concentration range of 1 to 600 to *μ*moles·mL^−1^. The LODs and LOQs were evaluated from the slope and residual standard deviation of the respective standard curves. An accuracy by spiking recovery test was assayed in which we prepared and injected in HPLC an amount of each GL, ranging from 1 to 100 moles·mL^−1^, followed by the respective determination of the amount found. The reference spiked samples were treated and analysed using the same procedure adopted for the plant samples. Recovery rate was calculated by comparing the amount of each GL detected in the spiked sample with the amount of each standard added. Instrumental precision was determined by replicate analysis of standard compounds (*n* = 7).

### 2.6. Statistical Analysis

SPSS for windows version 17.0 (SPSS-IBM, Orchard Road-Armonk, New York, USA) was used to calculate all statistical parameters. All experiments were done in triplicate.

## 3. Results and Discussion

### 3.1. Method Validation and Quality Assurance

The method was validated using calibration curves calibration curves and the analytical parameters tested were selectivity, linearity, precision, accuracy and recovery, limit of detection (LOD), and limit of quantification (LOQ). Two calibration curves for sinigrin and benzyl GL (the most common GLS standards and widely used as internal standard), with 7 points each, range from 1 to 600 to *μ*moles·mL^−1^. The LODs and LOQs were evaluated from the slope and residual standard deviation of the respective standard curves according the guidelines of ICH [24]. The accuracy by spiking recovery test was assayed in which we injected in HPLC an amount of each above compound in three levels of concentration: low (75 *μ*moles·mL^−1^), medium (150 *μ*moles·mL^−1^), and high (300 *μ*moles·mL^−1^) as recommended by ICH [24]. After that, we determined the respective amount of each GLS at each concentration injected applying the respective mathematical formula. Recovery rate was calculated by comparing the amount of each GLS detected in the spiked sample with the amount of each standard added. Instrumental precision was determined by replicate analysis of standard compounds (*n* = 7). SPSS for windows version 17.0 were used to calculate all statistical parameters (means, standard deviations, coefficient of variation, minimum and maximum, and correlation coefficient), and a *t*-test was used for determination of significant differences between the mean values. The method performance data are shown in Tables 2 and 3.

**Figure 2:**
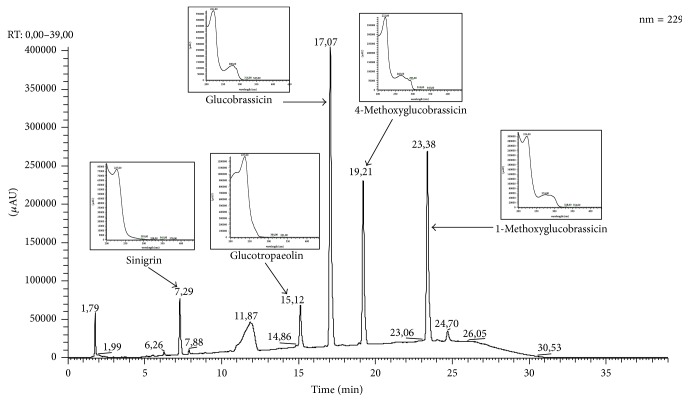
Typical chromatogram of GLS identified in Chinese cabbage hairy roots and respective UV spectra.

According to our results, the method showed a good linearity, with *R*^2^ > 0.99 which was highly significant (Table 2). The validity of assay was verified by ANOVA, and, according to it, there is linear regression and there is no deviation from linearity (*P* < 0.05). Although the recovery is sometimes quite difficult to assess due to the interference of other endogenous compounds in sample matrices, spiking standards during method development were done by spiking the standards into a blank and carrying through the extraction. Our results showed a variation of GLS recovery between 89.0 and 108.6% (Table 3), which according to literature [23, 25, 26] is acceptable and indicates good accuracy of the proposed HPLC method. In addition, the method showed good selectivity, since all GLS were well separated from each other with good resolution (Figure 2).

### 3.2. Profiling GLS of Chinese Cabbage Hairy Roots

HPLC separation of GLS from Chinese cabbage hairy roots is shown in Figure 2. The GLS peaks were identified according to their elution order and compared to the HPLC separation reported by previous works [27–31]. The HPLC analysis of known GLS composition from other research studies was used to help in the peak identification.

The compounds found were glucobrassicin, 4-methoxyglucobrassicin, and 1-methoxyglucobrassicin, also known as neoglucobrassicin (Table 4), all from indole type. The presence of sinigrin and benzyl GL, normally absent in Chinese hairy roots, is because they were added for validation process. Benzyl GL was used as internal standard (IS). Under the HPLC-DAD-UV/Vis conditions used in the current research, the glucosinolates sinigrin, benzyl GL, glucobrassicin, 4-methoxyglucobrassicin, and neoglucobrassicin were separated at RT of 7.29, 5.12, 17.07, 19.21, and 23.38 minutes, respectively (Table 4). The sinigrin and benzyl GL used as internal standard did not overlap with the endogenous GLS in Chinese cabbage hairy roots, which allow us to use the benzyl GL as IS when this is not present in the samples. The peaks of the three indole glucosinolates, glucobrassicin and its derivatives 4-methoxyglucobrassicin and neoglucobrassicin, were also clearly separated. However, in the cases in which this did not occur the gradient should be adapted, by decreasing or increasing of the rate of acetonitrile in the eluent or even the flow used. In addition, in the cases in which the GLS are lower than the expected, another extraction with more plant material should be done or, in alternative, an injection with higher volume when the amount of sample is too short. Compared to LC-MS-based methods reported in literature, which usually do not include sulfatase steps, our method is less labor intense and less expensive and thus it can be implemented routinely to separate and quantify GLS in plant sample matrices.

## 4. Conclusion

According to our results, the extraction and quantification method demonstrated a good linearity, a good precision, and a good selectivity.

Thus we can conclude that this method enables the detection and separation of commonly found GLS. In conclusion, this protocol provides an accessible method to extract and quantify glucosinolates in plant samples. This method, used to quantify GLS in Chinese cabbage hairy roots, is accurate, feasible, and very useful to maximize the amount of GLS extracted. The validation of this method has proved that it performs well and fits for its purpose. Nonetheless, the lack of some specific standards adds some level of uncertainty different sample preparation and instrument detection software will create additional differences when comparisons are made. Nonetheless, based on our results, the method is valid and can be extended to other plant or food matrices.

## Figures and Tables

**Table 1:** Response factors (Rf) of the most commonly desulfated glucosinolates detected at 229 nm, in plant extracts.

Structural class	Trivial name	Chemical name	Chemical formula & structure	Rf at 229 nm	Reference
Aliphatic GLS	Sinigrin	2-Propenyl	CH_2_=CH-CH_2_-	1.00	[21]
	Gluconapin	3-Butenyl	CH_2_=CH-CH_2_-CH_2_-	1.11	[21]
	Glucobrassicanapin	4-Pentenyl	CH_2_=CH-CH_2_-CH_2_- CH_2_-	1.154	[21, 22]
	Glucoerucin	4-Methylthiobutyl	CH_3_-S-CH_2_-CH_2_-CH_2_-CH_2_-	0.9	[23]
	Glucoiberin	3-Methylsulfinylpropyl	CH_3_-SO-CH_2_-CH_2_-CH_2_-	1.07	[21]
	Glucoraphanin	4-Methylsulfinylbutyl	CH_3_-SO-CH_2_-CH_2_-CH_2_-CH_2_-	1.07	[21]
	Glucoalyssin	5-Methylsulfinylpentyl	CH_3_-SO-CH_2_-CH_2_-CH_2_-CH_2_-CH_2_-	1.07	[21]
	Progoitrin	2-Hydroxy-3-butenyl	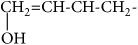	1.09	[21]

Indole GLS	4-Hydroxyglucobrassicin	4-Hydroxyindol-3-ylmethyl	4-HO-C_8_H_5_N-CH_2_-	0.28	[21]
	Glucobrassicin	Indol-3-ylmethyl	C_8_H_6_N-CH_2_-	0.29	[21]
	4-Methoxyglucobrassicin	4-Methoxyindol-3-ylmethyl	4-CH_3_O-C_8_H_5_N-CH_2_-	0.25	[21]
	Neoglucobrassicin	1-Methoxyindol-3-ylmethyl	1-CH_3_O-C_8_H_5_N-CH_2_-	0.20	[21]

Aromatic GLS	Gluconasturtiin	2-Phenylethyl	C_6_H_5_-CH_2_-CH_2_-	0.95	[21]
	Glucotropaeolin	Benzyl	C_6_H_5_-CH_2_-	0.95	[21]
	Sinalbin	4-Hydroxybenzyl	4-HO-C_6_H_4_-CH_2_-	0.4	[21]

Other desulphated undefined GLS				1.0	[21, 22]

**Table 2:** Linear range, correlation coefficient (*R*^2^), limit of detection (LOD), and limit of quantification (LOQ) of HPLC system used for glucosinolates determination.

Analyte	Linear range (*µ*moles·mL^−1^)	Regression equations	*R* ^2^ (*n* = 7)	LOD (*µ*moles·mL^−1^)	LOQ (*µ*moles·mL^−1^)
Sinigrin	1–600	*y* = 9690.2*x* + 244454	0.9949	1.8	5.3
Glucotropaeolin	1–600	*y* = 10517*x* + 40324	0.9998	2.0	5.9

**Table 3:** Accuracy of HPLC system used for glucosinolates determination.

Analyte	Prepared concentration (*µ*moles mL^−1^)	Measured concentration (*µ*moles mL^−1^)	Recovery (%)	Recovery (mean ± % RSD)
Sinigrin	300	325.9	108.6	108.6 ± 0.17
	300	326.5	108.8	
	300	325.4	108.5	
	150	152.5	101.7	101.3 ± 0.32
	150	151.7	101.1	
	150	151.8	101.2	
	75	66.9	89.3	89.0 ± 0.19
	75	66.7	88.9	
	75	66.7	88.9	

Glucotropaeolin	300	302.4	100.8	100.5 ± 0.41
	300	300.1	100.0	
	300	302.0	100.7	
	150	153.5	102.3	102.4 ± 0.03
	150	153.6	102.4	
	150	153.6	102.4	
	75	71.9	95.9	95.7 ± 0.31
	75	71.9	95.8	
	75	71.5	95.3	

**Table 4:** Glucosinolates identified in the current Chinese hairy roots. Respective retention time (Rt). Wavelengths of maximum absorption in the visible region by elution order and respective average content.^1^

GLS identified	Chemical formula & structure	Rt (min)	UV (nm)	HPLC-DAD-UV/Vis band (nm)	Average level (*µ*moles·100 g^−1^ dw)
Glucobrassicin	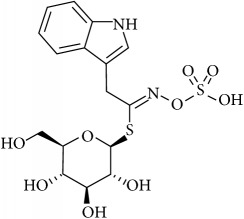	17.07	229	221	409.75
4-Methoxyglucobrassicin	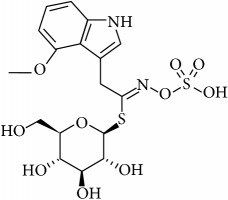	19.21	229	222	182.15
1-Methoxyglucobrassicin (neoglucobrassicin)	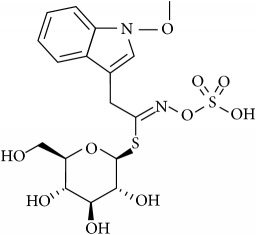	23.38	229	224	195.06

^1^Average value of glucosinolate content is expressed as mean of three replicates.
